# Effects of Di-Isononyl Phthalate (DiNP) on Follicular Atresia in Zebrafish Ovary

**DOI:** 10.3389/fendo.2021.677853

**Published:** 2021-06-14

**Authors:** Filipe G. Andrade Godoi, Isabel Forner-Piquer, Basilio Randazzo, Hamid R. Habibi, Fabiana L. Lo Nostro, Renata Guimarães Moreira, Oliana Carnevali

**Affiliations:** ^1^ Dipartimento Scienze della Vita e dell’Ambiente, Università Politecnica dele Marche, Ancona, Italy; ^2^ Departamento de Fisiologia, Instituto de Biociências, Universidade de São Paulo, Rua do Matão, Cidade Universitária, São Paulo, Brazil; ^3^ Department of Biological Sciences, University of Calgary, Calgary, AB, Canada; ^4^ Laboratorio de Ecotoxicología Acuática, IBBEA, CONICET-UBA & DBBE, FCEyN, Universidad de Buenos Aires, Buenos Aires, Argentina; ^5^ Istituto Nazionale Biostrutture Biosistemi, Consorzio Interuniversitario di Biosistemi e Biostrutture, Rome, Italy

**Keywords:** *Danio rerio*, endocrine disruption, phthalate, aquatic toxicology, Reproductive biomarkers, oxidative stress, follicular atresia

## Abstract

Di-isononyl phthalate (DiNP) is a plasticizer reported to elicit hormone-like activity and disrupt metabolism and reproduction in fish and other vertebrates. In general, phthalates have been used at high concentrations beyond reported environmental levels to assess their adverse effects on fish gonadal physiology. The present study exposed adult female zebrafish to a wide range of DiNP concentrations [0.42 µg L^−1^ (10^−9^ M), 4.2 µg L^−1^ (10^−8^ M), and 42 µg L^−1^ (10^−7^ M)] for 21 days. We evaluated gene expression profiles related to apoptosis, autophagy, and oxidative stress; DNA fragmentation (TUNEL assay: terminal deoxynucleotidyl transferase dUTP nick end labeling) and caspase activity (CAS3) were also examined. Exposure to 0.42 and 4.2 µg L^−1^ upregulated the genes coding for *tnfa* and *baxa*, *sod1, prkaa1*, respectively. CAS3 immunohistochemistry revealed a higher number of positive vitellogenic oocytes in ovaries exposed to 0.42 µg L^−1^. Subsequently, we examined the relationship between CAS3 signaling and DNA fragmentation. Accordingly, DNA fragmentation was observed in vitellogenic follicles of fish exposed to 0.42 and 4.2 μg L^−1^. Our results demonstrate that follicular atresia can occur after exposure to environmental levels of DiNP for 21 days, which may adversely affect the reproductive performance of female zebrafish in a non-monotonic manner.

**Graphical Abstract:**
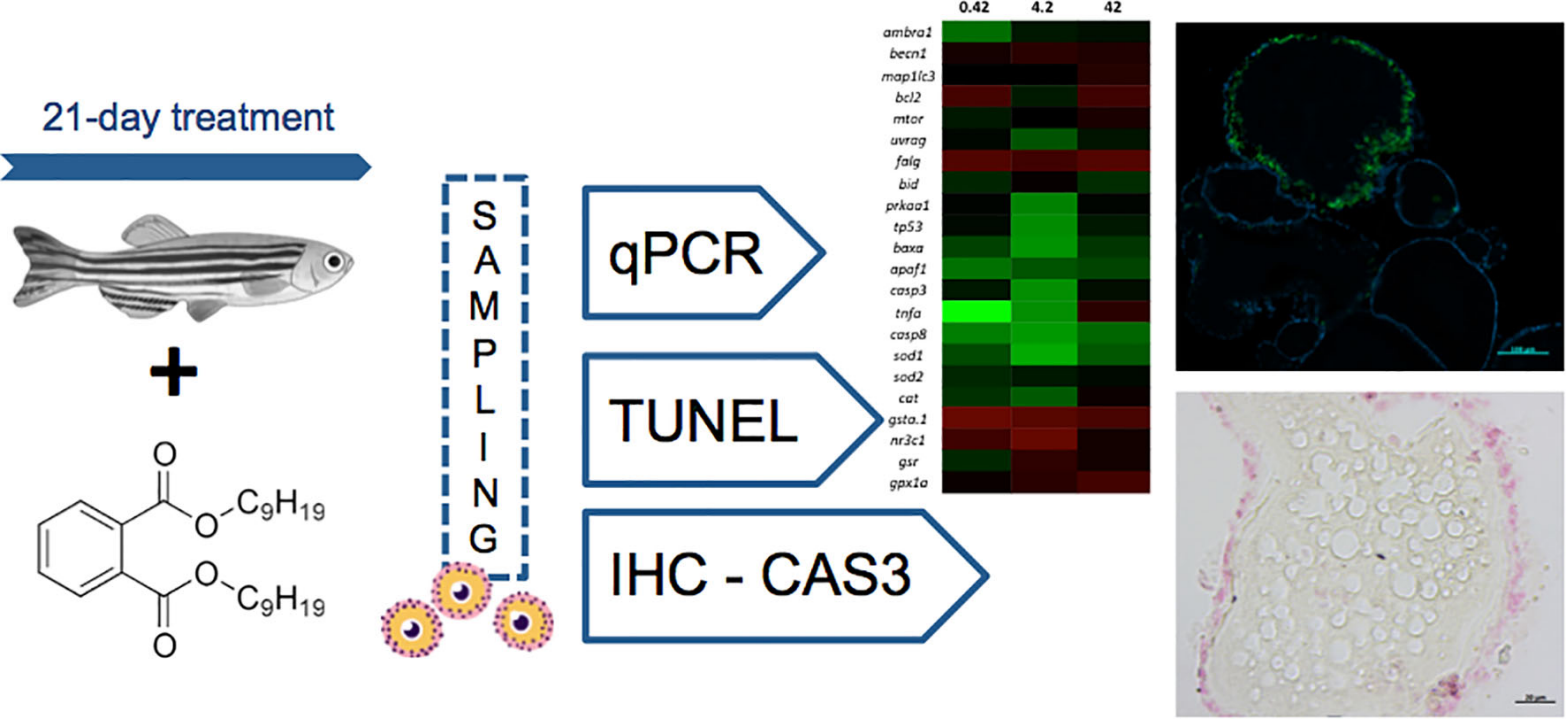


## Highlights

1. In adult female zebrafish, DiNP exposure elicited discrete gene expression modifications in the ovaries.
2. 21-day DiNP exposure enhanced caspase-3 activity in vitellogenic follicles.
3. Environmental concentrations of DiNP induced DNA fragmentation in follicular cells.

## Introduction

Approximately 6 million tons of phthalate esters are produced worldwide (1) and used primarily to improve the elasticity, strength, and durability of plastic items. The binding of phthalates to the plastic matrix is not covalent and can, therefore, migrate to the surroundings (2, 3). Currently, many phthalate esters, i.e., dimethyl phthalate (DMP), diethyl phthalate (DEP), di-n-butyl phthalate (DnBP), butyl benzyl phthalate (BBP), di-n-octyl phthalate (DnOP), and di(2-ethylhexyl) phthalate (DEHP), can be detected in aquatic ecosystems (4–6).

Among them, DEHP is one of the most studied phthalates in the aquatic environment (6, 7), and experimentally, the negative impacts of DEHP have been largely reported for teleost, i.e., disruption of oocytes growth and maturation, impairment of spermatogenesis, or hormonal dysfunction (8–11). Considering the major effects of DEHP on aquatic life, the di-isononyl phthalate (DiNP) was introduced in the plastic industry as a DEHP substitute (12) due to its similar applications (13), and also described as less harmful to reproduction when compared to other phthalates (14, 15). Currently, DiNP can be detected in wastewater, soil (7, 16), and surface waters with concentrations ranging from 0.23 to 85 µg L^−1^ (see *Materials and Methods*).

Recent studies conducted in our laboratory revealed that in zebrafish, DiNP altered the gonadal endocannabinoid system, oocyte growth, and oocyte maturation (17, 18). The observed reproductive effects of DiNP indicate that this phthalate is an endocrine disrupting chemical (EDC), capable of interfering with the reproductive physiology and, therefore, requesting further studies to decipher the impacts of phthalates toxicity (19, 20).

One of the reproductive mechanisms in female gonads is the follicular atresia, a process based on follicular reabsorption throughout an interplay between apoptosis and autophagy, ensuring reproductive viability of the follicles in the ovaries (21, 22), and also tightly regulated by hormones (23). Under normal conditions, these processes regulate follicular atresia during yolk reabsorption and cell death (22), but such processes might be disrupted following unfavorable environmental conditions leading to the progression of atresia (24).

The role of autophagy and apoptosis during follicular atresia depends on key molecular components that regulate each process (25). In this context, the caspase-dependent (CAS) pathway is among the main mechanisms driving apoptosis and programmed cell death in the ovary. Extrinsically, CAS3 is activated by CAS8, which is recruited after the expression of the tumor necrosis factor receptor (TNFR) family, TNF-related apoptosis-inducing ligand (TRAIL) receptors, and FAS receptors (25). Apoptosis can also be initiated through an intrinsic mitochondria-dependent pathway *via* activation of CAS9 and CAS3, involving the mitochondrial outer membrane permeabilization (MOMP) and p53 tumor suppressor (25). It should be noted that autophagy is essential for cellular homeostasis during normal stress conditions. The process of autophagy involves the formation of autophagosomes, which are activated by several molecules, such as mTOR (mammalian Target of Rapamycin) or beclin1 (26). These processes involve a number of key molecules that can occur simultaneously and can be disrupted in response to environmental stressors and abnormal physiological conditions (25).

To better understand this process, we examined the effects of exposure to environmentally relevant concentrations of DiNP on zebrafish ovarian function, using different molecular and cellular approaches to further investigate follicular atresia through apoptosis and autophagy.

## Material And Methods

### Experimental Design

Adult female zebrafish (*Danio rerio*, AB wild-type strain, 1-year-old, 0.6 ± 0.15 g) were maintained in 100-L glass aquaria with oxygenated water under controlled conditions (28.0 ± 0.5°C; 14/10 h of light/dark period). Fish were fed twice a day with a 1:1 mixture of adult zebrafish complete diet (Zeigler Bross., Inc.) and live brine shrimp *nauplii*. Females (25 per group, in duplicate) were exposed for 21 days to three nominal concentrations of DiNP (Sigma-Aldrich; 99% purity): 0.42 µg L^−1^ (10^−9^ M); 4.2 µg L^−1^ (10^−8^ M); 42 µg L^−1^ (10^−7^ M) and a control (CTL) free of DiNP. The concentration range used was based on our previous studies (17, 18) and information from reported environmental concentrations of DiNP, ranging from 0.52 µg L^−1^ in European waters (27), 0.15 to 1.98 µg L^−1^ in European rivers (28), 21 and 70 µg L^−1^ in urban run-off (29), and 85 µg L^−1^ in urban storm-water (30).

In the present study, DiNP concentrations in water were confirmed using gas chromatography-mass spectrometry (GC-MS). Because of its poor water solubility, DiNP was diluted in 1 ml of absolute EtOH and then, added to each aquarium. EtOH concentration (0.001% v/v) was below the activity threshold reported previously (31, 32).

After 21 days, fish were euthanized with an overdose of buffered methane sulfonate MS-222 (300 mg L^−1^) (Sigma-Aldrich). Ovarian samples for molecular analysis were immediately frozen at −80°C with dry ice. Samples for TUNEL and CAS3 techniques were fixed in Bouin’s solution (Bio-Optica) overnight, then rinsed in 70% EtOH and stored at 4°C until needed for further analysis. Experiments were conducted in accordance with the guidelines on the care and use of fish in research, teaching, and testing from the Canadian Council on Animal Care (2005), as well as being in compliance with the University of Calgary animal care protocol.

### RNA Extraction and cDNA Synthesis

RNA extraction, assessment of the RNA quality/quantity, and cDNA synthesis were performed using the whole ovary following (33). Briefly, RNA was extracted with TRIzol^®^ reagent (Invitrogen) followed by chloroform disaggregation. RNA was precipitated with isopropanol and washed twice with absolute ethanol. Samples were treated with DNAse, and RNA quantification was determined by spectrophotometry. The quality of mRNA was assessed by electrophoresis in 1% agarose gel.

### Real-Time PCR (RT-qPCR)

The relative quantification of gene transcripts was conducted using SYBR Green dye-based detection in a CFX iCycler thermal cycler (Bio-Rad). All samples were analyzed in duplicates. Each reaction contained: 1 μl of diluted (1/10) cDNA, 5 μl of 2× SYBR Green PCR Master Mix (Bio Rad), 0.1 μl of both forward and reverse primers, and 3.8 μl of milliQ water with a final volume of 10 μl per well. The reference genes used were *rplp0* (Ribosomal Protein Large P0) and *rplp13* (Ribosomal Protein Large P13). A list of the used primers is provided in **Table 1**. Data were analyzed using iQ5 Optical System version 2.0 (Bio-Rad), including Genex Macro iQ5 Conversion and Genex Macro iQ5 files (33).

**Table 1:** List of primers for Real Time qPCR analysis.

Type	Name	Abb.	Genbank accession number	FORWARD (5′-3′)	REVERSE (5′-3′)	Tm (°C)
Housekeeper	Ribosomal Protein Large P0	*rplp0*	NM_131580.2	CTGAACATCTCGCCCTTCTC	TAGCCGATCTGCAGACACAC	60
Housekeeper	Ribosomal Protein Large P13	*rplp13*	NM_212784.1	TCTGGAGGACTGTAAGAGGTATGC	AGACGCACAATCTTGAGAGCAG	59
Autophagy	Beclin 1, autophagy related	*becn1*	NM_200872.1	GGACCACTTGGAACAACT	CCGAAGTTCTTCAGTGTCCATC	60
Autophagy	Microtubule-associated protein 1 light chain 3	*map1lc3c*	NM_200298.2	GAGAAGTTTTTGCCGCCTCT	ACCTGTGTCCGAACATCTCC	60
Apoptosis	BCL2 associated X, apoptosis regulator a	*baxa*	NM_131562.2	GGCTATTTCAACCAGGGTTCC	TGCGAATCACCAATGCTGT	60
Apoptosis	Apoptotic peptidase activating factor 1	*apaf1*	NM_131608.1	TTCTACAGTAAACGCCCACC	TATCTAGTATTTCCCCATATTCC	60
Apoptosis	Caspase 3	*casp3*	NM_131877.3	CCGCTGCCCATCACTA	ATCCTTTCACGACCATCT	60
Apoptosis	BCL2 apoptosis regulator	*bcl2*	NM_001030253.2	CCTTCAATAAAGCAGTGGAGGAA	CGGGCTATCAGGCATTCAGA	60
Apoptosis	Tumor necrosis factor *a*	*tnfa*	NM_001002184.1	TTGTGGTGGGGTTTGATG	TTGGGGCATTTTATTTTGTAAG	60
Autophagy	Mammalian Target of Rapamycin	*mtor*	NM_001077211.2	AACCTACTGCCTCGACTTGC	CTCACAGCCACCACCAGTAG	60
Autophagy	UV radiation resistance associated gene	*uvrag*	NM_201069.1	GCGAGTGGAGGAGAGRGTATG	GCCGTGAGACCTCTTCAATC	60
Apoptosis	Fas ligand (TNF superfamily, member 6)	*faslg*	NM_001042701.2	AGCCCGAGTCGAGATGAAGA	CAACTTGTTTCTGTGGGGCG	60
Autophagy	BH3 interacting domain death agonist	*bid*	NM_001079826.1	GCAGCAGCCAAAGAGTTTAAGAAGGAG	AGGTGTGCGTTCACAAACAGTCTTCA	60
Autophagy	Protein kinase AMP-activated, alpha 1 catalytic	*prkaa1*	NM_001110286.1	GCACCTTCCACGCCTCCGATT	CCAGAGAGCCTTCCGCCACTTTAC	60
Apoptosis	Caspase 8	*casp8*	MG958000.1	GTTTTGGGCACAGATGGTAA	TACTGTGGCCATTCCGATCA	60
Oxidative stress	Superoxide dismutase 1	*sod1*	AY195857	CCGGACTATGTTAAGGCCATCT	ACACTCGGTTGCTCTCTTTTCTCT	60
Oxidative stress	Superoxide dismutase 2	*sod2*	NM_199976.1	GTCTGTTGGTTGGTCGCTTG	GCACCTAACAGGGGGTTGAA	60
Oxidative stress	Catalase	*cat*	NM_130912.2	AGGGCAACTGGGATCTTACA	TTTATGGGACCAGACCTTGG	60
Oxidative stress	Glutathione S-transferase, alpha tandem duplicate 1	*gsta.1*	NM_213394.1	TTGAGGAAAAGGCCAAAGTG	AACACGGCCTTCACTGTTCT	60
Endocrine	Glucocorticoid receptor	*nr3c1*	NM_001020711.3	CGCCTTTAATCATGGGAGAA	AGACCTTGGTCCCCTTCACT	58
Oxidative stress	Glutathione reductase	*gsr*	NM_001020554.1	GAACGGGGTCATATCGTGGT	TGGAGACCGACCACCTTTTC	60
Oxidative stress	Glutathione peroxidase 1a	*gpx1a*	NM_001007281.2	ACCTGTCCGCGAAACTATTG	TGACTGTTGTGCCTCAAAG	60

### Terminal Deoxynucleotidyl Transferase dUTP Nick End Labeling (TUNEL) Assay

Ovaries were processed according to Randazzo et al. (34). Briefly, after dehydration in graded EtOH solutions, samples were clarified in xylene and embedded in paraffin. Sections (5 μm) obtained using a microtome (Leica RM2125 RTS) were placed on gelatinized slides and processed following the manufacture protocol (Roche Diagnostics GmbH). Next, sections were deparaffinized in xylene, rehydrated, and post-fixed using 4% paraformaldehyde (PFA). Positive control was performed without the PFA. All slides were rinsed (15 min) with proteinase K to deactivate free enzymes in a humidified chamber. Then, the slides were treated with TdT buffer 1× for 30 min, followed by overnight incubation at 4°C with the reaction mixture (10 μl TdT buffer 5×, 5 μ CoCl_2_, 0.5 μl digoxigenin-11-UTP, 1.5 μl terminal transferase enzyme, and 33 μl PBS per sample). Next, the slides were washed with EDTA (1 mM in PBS), blocked with sheep serum, and incubated with anti-digoxigenin AP (1:5.000) for 90 min at room temperature (RT). Finally, the staining solution was freshly prepared by dissolving one Fast Red pill in 2 ml of buffer (5 ml Tris HCl 1 M, 50 μl Tween 20, 45 ml deionized H_2_O, pH 8.2) to mark the reaction. The sections were observed under a Zeiss Axio Imager.M2 coupled with a high-resolution Zeiss Axiocam 105 color camera.

### Caspase 3 Immunohistochemistry Assay (CAS3)

Deparaffinized slide sections (5 μm) were rehydrated and treated with citrate buffer (10 mM sodium citrate, 10 mM citric acid, 0.05% Tween 20, pH 6.0) for the antigen retrieval. Next, endogenous peroxidases were inactivated using 3% H_2_O_2_ at RT for 5 min. Then, to block non-specific bindings, slide sections were treated with 40% calf serum (Sigma-Aldrich). After the blocking, sections were incubated overnight in a humid chamber at 4°C with the primary cleaved CAS3 antibody from rabbit (1:200) (Abcam, 13847) recognizing a cleaved form of Caspase 3 (~17 kDa). The next day, sections were rinsed with PBS buffer (pH 7.5) for 15 min and incubated with the secondary antibody IgG (Alexa Fluor 488) goat Anti-Rabbit diluted at 1:500 (Abcam, 150077) at RT for 2 h. Subsequently, the slides were mounted with Fluoroshield mounting medium with DAPI (Abcam, 104139) for nuclei staining. Sections were observed under a Zeiss Axio Imager.M2 microscope, and images were acquired with a high-resolution Zeiss Axiocam 506 monochromatic camera. Images were processed with ZEN 2.3 lite software (Zeiss Microscopy GmbH).

The relative frequency of atretic follicles and vitellogenic follicles (focusing on stages III and IV) with CAS3-positive reaction were evaluated as described previously (35, 36).

### Statistical Analysis

The data were analyzed with one-way ANOVA when fulfilled the conditions for applying a parametric test followed by Dunnett’s *post hoc* analysis. When data did not accomplish those conditions, Kruskal-Wallis non-parametric test was used. All statistical procedures were run using *GraphPad Prism 6*. The results are reported as mean ± standard error of the mean (SEM). Statistical significance was set at p < 0.05. Heatmap design was conducted using R software. Briefly, gene expression was normalized and standardized using z-score, and then, the mean was clustered with Euclidean distance matrix method. The results were expressed as a hierarchical clustering dendrogram followed by the heatmap plot. The relative frequency of CAS3 signal was assessed using a generalized linear model (glm) in R software (37).

## Results

### Discrete Dose-Dependent Gene Expression Deregulations in Response to DiNP

**Figure 1:**
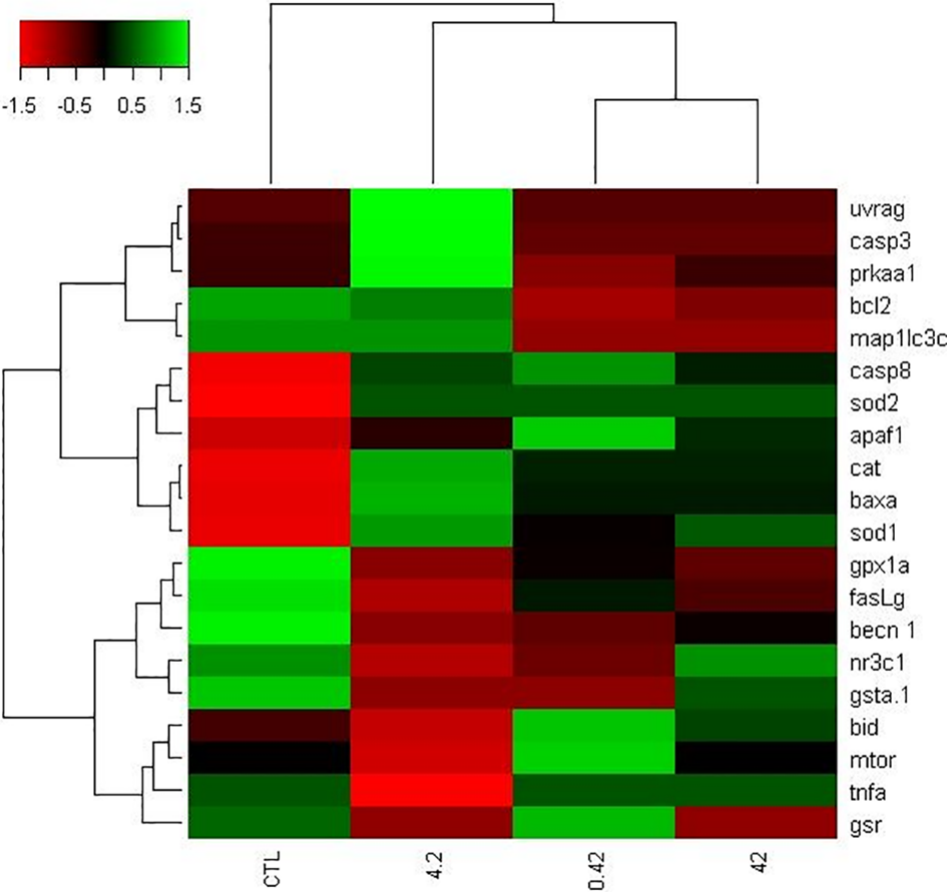
Gene expression heatmap (autophagy, apoptosis and oxidative stress) from zebrafish ovaries exposed to DINP. Color code to represented upregulated genes (green) and downregulated genes (red). Both rows (genes) and columns (treatments) were grouped by hierarchical clustering.

We examined the effects of DiNP on the transcriptomic profile to unravel candidate markers for apoptosis, autophagy, and oxidative stress in the *D. rerio* ovaries. Results are summarized in **Table 2**. Genes coding for the autophagic pathway (*becn1, map1lc3c, mtor, uvrag, bid*) were not significantly altered, except for *prkaa1* which displayed an upregulation in the 4.2 μg L^−1^ group. Additionally, 0.42 and 4.2 μg L^−1^ ovaries evidenced increased mRNA levels for *baxa* and *tnfa*, genes related to the apoptotic pathway. The gene coding for *sod1* was the only oxidative stress marker showing an upregulated expression in the 4.2 μg L^−1^ group. Gene expressions were visually described as a heatmap (**Figure 1**).

**Table 2:** Transcriptional effects of DiNP on the zebrafish ovary.

GENE	CTL	0.42 μg L^-1^	4.2 μg L^-1^	42 μg L^-1^
*becn 1*	2.09 ± 0.44	1.93 ± 0.17	1.77 ± 0.14	1.84 ± 0.34
*map1lc3c*	1.42 ± 0.05	1.43 ± 0.06	1.43 ± 0.06	1.22 ± 0.07
*baxa*	1.73 ± 0.20	2.18 ± 0.19	2.74 ± 0.28**	2.10 ± 0.31
*apaf1*	1.65 ± 0.20	2.39 ± 0.28	2.20 ± 0.36	2.10 ± 0.30
*casp3*	1.88 ± 0.32	2.05 ± 0.25	2.59 ± 0.40	1.99 ± 0.19
*bcl2*	2.02 ± 0.28	1.47 ± 0.15	2.22 ± 0.50	1.52 ± 0.17
*tnfa*	7.24 ± 4.07	47.13 ± 16.89*	11.29 ± 2.92	6.13 ± 2.96
*mtor*	2.46 ± 0.41	2.68 ± 0.17	2.46 ± 0.39	2.21 ± 0.20
*uvrag*	3.62 ± 0.69	3.71 ± 0.37	4.81 ± 0.64	3.90 ± 0.56
*fasLg*	3.23 ± 0.74	2.21 ± 0.20	2.45 ± 0.47	2.18 ± 0.41
*bid*	1.29 ± 0.18	1.48 ± 0.13	1.26 ± 0.09	1.52 ± 0.12
*prkaa1*	1.72 ± 0.24	1.75 ± 0.10	2.60 ± 0.12**	1.75 ± 0.13
*casp8*	1.47 ± 0.18	2.20 ± 0.37	2.35 ± 0.48	2.07 ± 0.31
*sod1*	1.65 ± 0.22	2.12 ± 0.16	2.74 ± 0.15**	2.21 ± 0.22
*sod2*	1.25 ± 0.06	1.44 ± 0.12	1.37 ± 0.10	1.31 ± 0.10
*cat*	2.34 ± 0.38	2.79 ± 0.23	3.16 ± 0.46	2.22 ± 0.20
*gsta.1*	17.60 ± 2.91	10.52 ± 3.09	11.62 ± 3.96	12.71 ± 1.14
*nr3c1*	3.47 ± 1.09	2.65 ± 0.42	2.10 ± 0.56	3.24 ± 0.75
*gsr*	1.83 ± 0.28	2.11 ± 0.42	1.51 ± 0.05	1.71 ± 0.27
*gpx1a*	2.08 ± 0.15	1.99 ± 0.11	1.75 ± 0.28	1.54 ± 0.13

Data were normalized against the expression of rplp0 and rplp13.

Data are reported as mean dCt ± SEM.

Asterisk superscript (*) indicates significant differences between control group (CTL) and DiNP treatment (one-way ANOVA, Dunnett’s multiple comparison test, *p < 0.05, **p < 0.01).

Genes: becn 1, Beclin 1 multifactorial protein; map1lc3c, microtubule-associated protein 1 light chain 3; bax, bcl2 associated X; apaf1, apoptotic peptidase activating factor 1; casp3, caspase 3; bcl 2, BCL2 apoptosis regulator; tnfa, tumor necrosis factor a; mtor, mammalian target of rapamycin; uvrag, UV irradiation resistance-associated tumor suppressor; faslg, Fas ligand; bid, BH3 interacting domain death agonist; prkaa1, protein kinase AMP-activated alpha 1 catalytic; casp8, caspase 8; sod1, superoxide dismutase 1; sod2, superoxide dismutase 2; cat, catalase; gst a1, glutathione transferase; nr3c1, glucocorticoid receptor gene; gsr, glutathione reductase; gpx1a, glutathione peroxidase.

### Impact of DiNP on Follicle DNA Fragmentation

**Figure 2:**
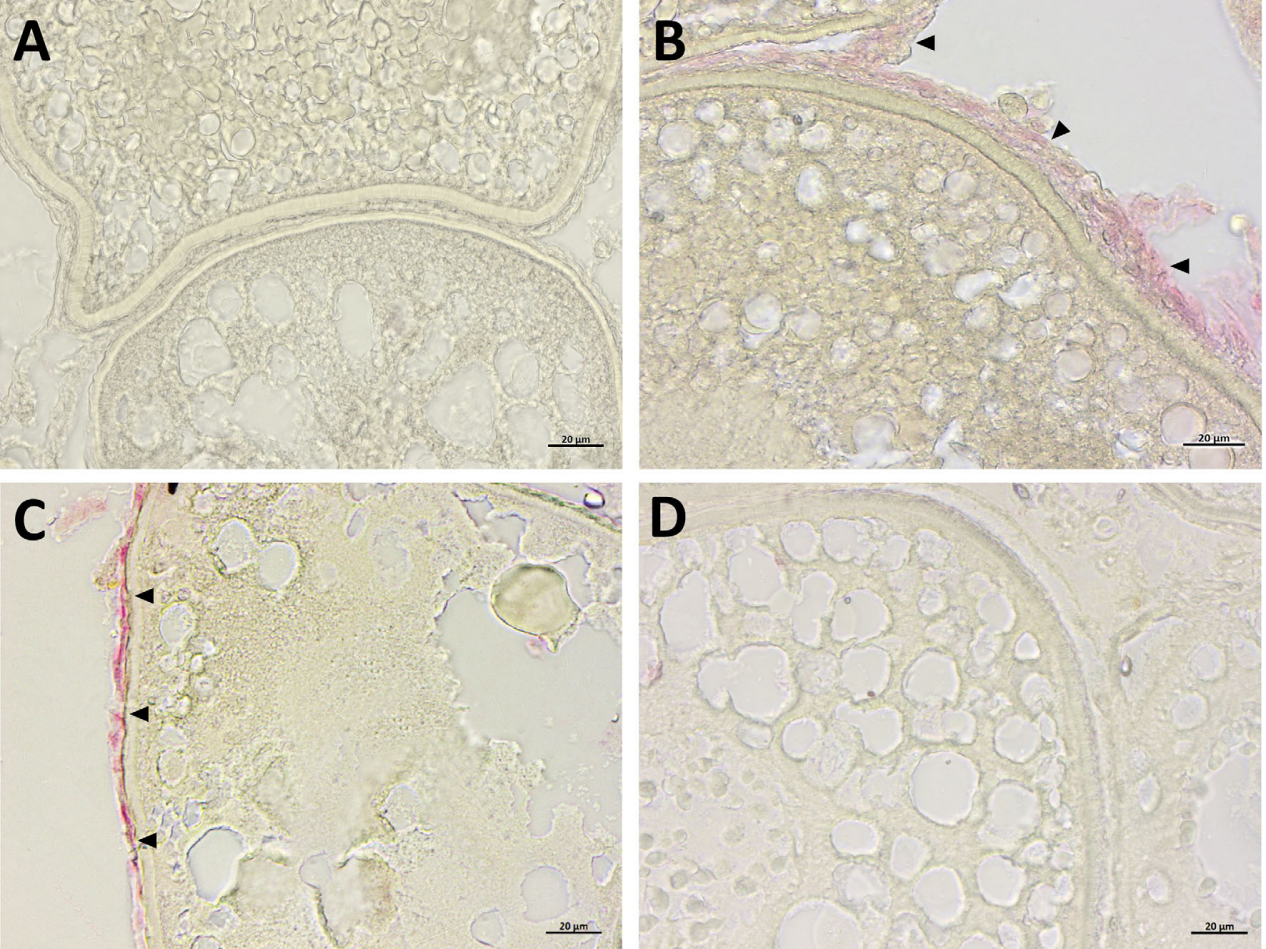
TUNEL assay in ovaries sections from zebrafish exposed to DiNP for 21 days. Positive TUNEL reaction stained in red (arrowheads). CTL **(A)**; 0.42 μg L^−1^ DiNP **(B)**; 4.2 μg L^−1^ DiNP **(C)**; 42 μg L^−1^ DiNP **(D)**. Scale bar: 20 μm.

To further investigate the potential apoptotic effects, TUNEL assay was used to evaluate DNA fragmentation. Positive DNA fragmentation label (**Figure 2**, red staining) was observed in follicular cells of atretic and vitellogenic follicles of zebrafish exposed to 0.42 μg L^−1^ (**Figure 2B**) and 4.2 μg L^−1^ (**Figure 2C**). No reaction was evidenced following exposure to the highest DiNP concentration (**Figure 2D**), nor was in the CTL group (**Figure 2A**).

### DiNP Differentially Affects CAS3 Expression in Ovarian Follicle Cells

**Figure 3:**
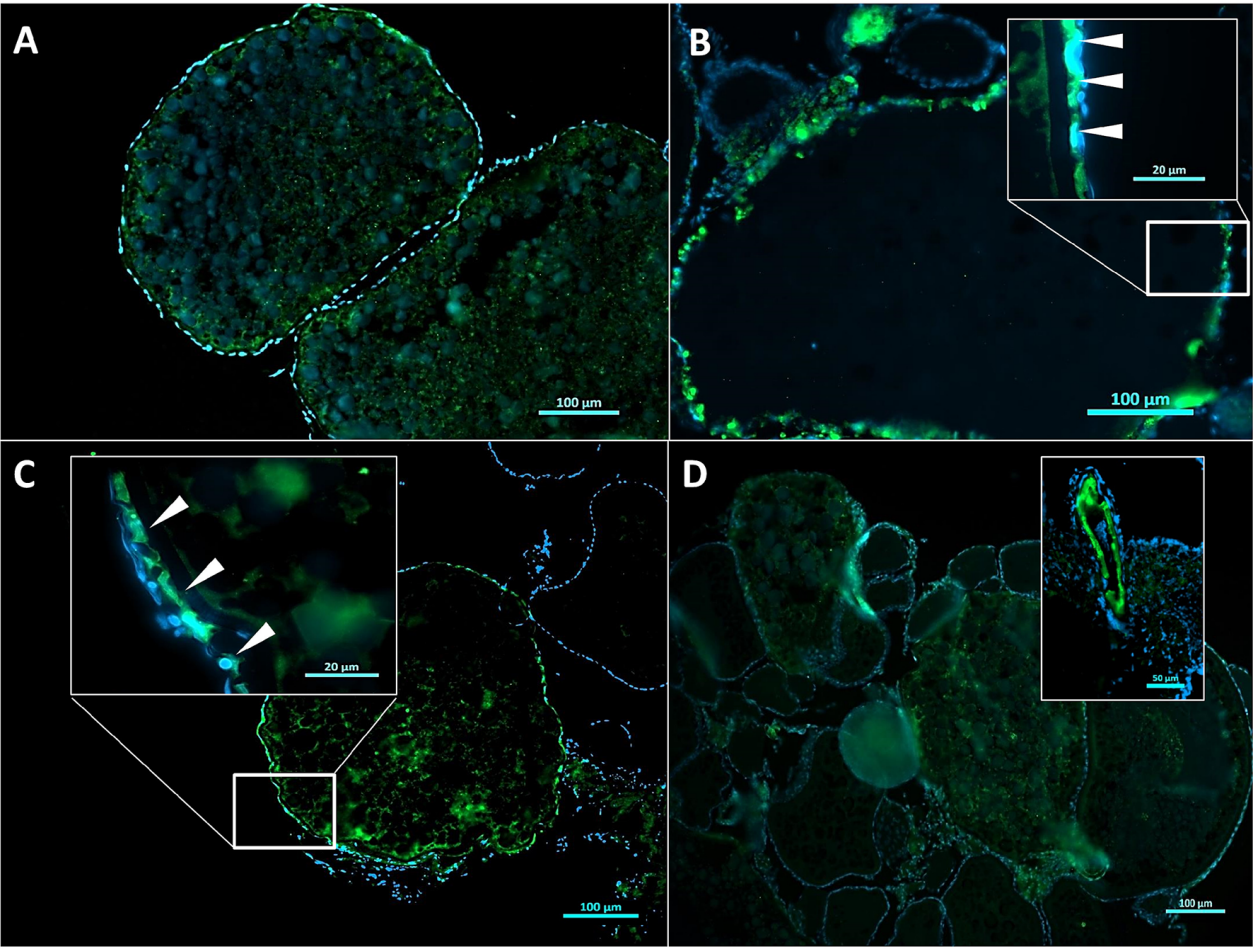
Immunolocalization of CAS3 (ir-CAS3) in ovaries cross-sections from control and exposed zebrafish for 21 days to DiNP. CTL **(A)**, 0.42 μg L^−1^ DiNP **(B)**, 4.2 μg L^−1^ DiNP **(C)**, 42 μg L^−1^ DiNP **(D)**. CAS3 signal in green (arrowheads) and DAPI nuclei counterstaining in blue. Scale bar: 100 μm; insert in **(B, C)** 20 μm; insert in **(D)** 50 μm.

Positive immunoreaction for CAS3 (ir-CAS3; **Figure 3**) was identified within the follicle cells of vitellogenic and atretic oocytes in CTL group (**Figure 3A**). Similarly, ir-CAS3–positive cells were shown in vitellogenic oocytes and atretic from 0.42 μg L^−1^ (**Figure 3B**), 4.2 μg L^−1^ (**Figure 3C**), and 42 μg L^−1^ (**Figure 3D**) DiNP groups. Inserts in **Figures 3B, C** evidenced the ir-CAS3 in follicular cells (white arrowheads).

Specifically, quantification of the relative frequency of positive vitellogenic follicles stage III (VF III), vitellogenic follicles stage IV (VF IV), as well as atretic follicles (AF), displayed a higher frequency of ir-CAS3 in VF IV from 0.42 μg L^−1^ DiNP ovaries compared to females CTL group (results provided in **Table 3**).

**Table 3:** Relative frequency of CAS3 signal on vitellogenic follicles stage III (VF III), vitellogenic follicles stage IV (VF IV) and atretic follicles (AF) in zebrafish ovaries exposed to DiNP.

	VF III	VF IV	AF
CTL	14.29%	42.86%	42.86%
0.42 μg L^−1^	33.33%*	38.89%	27.78%
4.2 μg L^−1^	13.33%	60.00%	26.67%
42 μg L^−1^	16.67%	50.00%	33.33%

Asterisk superscript (*) indicates significant differences between control group (CTL) and DiNP treatments (glm test, *p < 0.05).

## Discussion

The present study extends the knowledge about the effects of DiNP exposure on zebrafish ovarian follicular apoptosis by providing novel information on discrete gene expression modifications, DNA fragmentation, and increased ir-CAS3 in follicular cells.

From an experimental standpoint, previous studies demonstrated the ability of phthalates or other xenobiotics, such as DEHP or bisphenol A (BPA), to exacerbate atresia in cultured mouse antral or zebrafish oocytes, respectively (35, 38). In the case of zebrafish, low concentrations of BPA induce atresia by accelerating the vitellogenic phase III progression, resulting in abnormally enlarged phase IV follicles. It should be noted that a previous study from our laboratory demonstrated that zebrafish exposed to DiNP led to a reduction in the number of vitellogenic oocytes (18), which is consistent with the observed higher frequency of ir-CAS3 signal in the DiNP VF III. There is evidence that apoptosis is associated with follicle atresia (23), impairing ovarian function (24). In addition, our previous study demonstrated that exposure to DiNP downregulated genes involved in steroidogenesis, oocyte growth, and maturation (18).

While the RT-qPCR approach provides useful information on gene expression patterns following xenobiotic exposures, our results demonstrated that exposure to environmental concentrations of DiNP increased transcript abundance for a limited number of genes, including *baxa, tnfa, prkaa1* and *sod1*. It is likely that the apoptotic activation (extrinsic and intrinsic) involved changes in *baxa* and *tnfa* expression. On one hand, the extrinsic apoptotic pathway depends on TNF family for the activation of CAS cascade (25), and on the other, *baxa* is an internal signal for supporting the release of pro-apoptotic proteins and, consequently, causing DNA fragmentation and production of reactive oxygen species (ROS) (39). Rats exposed to di-n-butyl phthalate (DBP) and DiNP displayed increased TNF-α and BAX in the brain and an alteration of SOD activity, indicating that DBP and DiNP may also trigger neuroinflammation and apoptotic activation in the brain (40, 41). Similarly, rats exposed to 400 μM of DEHP resulted in amplified mitochondria-ROS mediated apoptosis in granulosa cells and enhanced ovarian expression of apoptotic genes including *baxa* and *cas3* (20).

We here reported that exposure to DiNP altered CAS3 signal and DNA fragmentation in follicular cells and that DiNP could induce apoptotic processes in the zebrafish ovary, accordingly with previous observations in rats treated with DiNP and other phthalate congeners (20, 40, 41). Furthermore, there is evidence that plasticizers, in general, exert deleterious effects by inducing oxidative stress in rodent ovary, dysregulating steroid production and activating cell death mechanisms (42).

Studies with phthalates are mainly conducted in mammals with non-realistic concentrations, presumably due to the phylogenetic proximity to humans. Since these compounds are abundantly present in the aquatic ecosystems (27), there is a need to study their effects on aquatic species (43) A recent study from our laboratory evidenced a decline in oocyte growth and maturation following DiNP exposure in zebrafish (18). Accordingly, we here reinforced these data by providing evidence on the ability of DiNP to induce follicle apoptosis. However, it should be noted that we used a heterologous polyclonal antibody raised against the cleaved form of human Caspase 3 (~17 kDa). We here acknowledge that this may constitute a limitation of interpreting immunohistochemistry data since we cannot rule out the remote possibility of cross-reactivity with the pro-caspase 3 (37 kDa). Thus, supporting the validity of our findings, other studies have previously demonstrated the specificity of this antibody for the active form of zebrafish CAS3 (44–48).

In addition, there is a crosstalk mechanism between autophagy and apoptotic processes in ovarian follicular atresia in fish species, highlighting the importance of maintaining a balance between the atresia/autophagy and folliculogenesis (21, 22). In this line, although we did not observe significant alterations in the transcripts of genes coding for autophagy (*becn1, map1lc3c, mtor, uvrag, bid*), any change in the proapoptotic factors could alter the homeostatic balance of follicular development and demise. After DiNP exposure, the autophagic gene coding for *prkaa1* was the only upregulated marker, indicating possible metabolic stress (49), and further unfavorable physiological conditions.

Finally, our data outlined a non-monotonic mode of action for DiNP (17, 18, 42), where lower concentrations seem to disrupt the ovarian function. This is not an unusual characteristic of environmental contaminants acting as endocrine disrupting chemicals (EDCs), such as phthalates, which can interact with multiple receptor-mediated endocrine and metabolic pathways (50–52). Considering the non-monotonic nature of DiNP in fish, exposure to low concentrations of DiNP and other phthalates found in the aquatic environment may contribute to a vulnerable reproductive condition with adverse consequences on reproductive success of aquatic organisms.

## Conclusion

We provide novel data highlighting the capacity of DiNP to induce apoptotic processes, unbalancing the follicular atresia and consequently, impairing the reproductive performance of *Danio rerio* females in a non-monotonic way. However, the present research leaves several significant questions that might be further examined. Foremost is the significance of the data obtained in zebrafish with DiNP to other aquatic species and other phthalate congeners exposure at it can occur due to the ubiquity of phthalates in aquatic ecosystems. From a pathophysiological standpoint, the implication of DiNP on the dynamic of folliculogenesis/oogenesis and the underlining mechanism on atresia remain to be fully understood and should be included in follow-up studies. Our data outlined discrete gene expression changes at low DiNP concentrations, thus, further analysis are required targeting those specific genes that may be importantly implicated in the phenotypes here reported.

In conclusion, our results provide a set of novel data on the dose-dependent effects of DiNP to the zebrafish ovary, suggesting that apoptotic biomarkers may be used as a relevant tool to evaluate the impacts of EDCs on the folliculogenesis of aquatic species.

## Data Availability Statement

The raw data supporting the conclusions of this article will be made available by the authors, without undue reservation.

## Ethics Statement

The animal study was reviewed and approved by Canadian Council on Animal Care (2005) as well as in compliance with the University of Calgary protocol.

## Author Contributions

Conceptualization: OC, HRH, FLN, and RM. Data curation: FG and IFP. Formal analysis: FG, IFP, and BR. Funding acquisition: OC. Investigation: FG and IFP. Methodology: FG, IFP, and BR. Project administration, resources, supervision, and validation: OC. Visualization: OC. Roles/writing—original draft: FG, OC, and HRH. Writing—review and editing: FG, IFP, OC, FLN, HRH, RM, BR. All authors contributed to the article and approved the submitted version.

## Funding

This work was supported by *Progetti di Rilevante Interesse Nazionale* (PRIN) 2010–2011 (prot 2010W87LBJ) to OC, and by *Fundação de Amparo à Pesquisa do Estado de São Paulo* (FAPESP; Processes: 2018/10495-0; 2017/07139-5, 2017/11530-1 and 2014/16320-7) to FG and RM.

## Conflict of Interest

The authors declare that the research was conducted in the absence of any commercial or financial relationships that could be construed as a potential conflict of interest.
